# Modifications of Cytochrome *c* by Retinoic Acid Play a Crucial Role in Mitochondrial Dysfunction of Triple-Positive Human Breast Cancer Cells: Raman Spectroscopy and Imaging Study

**DOI:** 10.1021/acsomega.5c00393

**Published:** 2025-06-24

**Authors:** Halina Abramczyk, Monika Kopeć, Jakub Surmacki

**Affiliations:** Laboratory of Laser Molecular Spectroscopy, Department of Chemistry, Institute of Applied Radiation Chemistry, 49584 Lodz University of Technology, Wróblewskiego 15, 93-590 Łódź, Poland

## Abstract

Conventional assays for the assessment of the receptors on the surface and inside specific organelles in human breast cancer cells include immunohistochemistry (IHC) and in situ hybridization (ISH), both of which have limitations. We propose a novel Raman method to monitor modifications such as the redox status of cytochrome *c* and phosphorylation triggered in cells by retinoic acid. We showed that Raman imaging provides an effective assay for detecting the redox status of cytochrome *c* and tyrosine kinase activity in specific cell organelles of human triple-positive breast cancer cells MCF-7 upon incubation with retinoic acid. Therefore, in contrast to existing analytical technologies, Raman imaging can detect the full extent of cytochrome *c* localization and tyrosine activity inside and outside specific organelles. We found that retinoic acid has a spectacular impact on mitochondrial functional activity in cancer cells. Abnormal retinoic acid signaling in the mitochondria, cytoplasm, lipid droplets, endoplasmic reticulum, and nucleus was monitored by Raman signals at 1582 cm^–1^, 1616 cm^–1^, 3058 cm^–1^, and 3072 cm^–1^ in mitochondria, cytoplasm, lipid droplets, endoplasmic reticulum, and nucleus. In human breast cancer cells, it was found that the balance between Fe^3+^/Fe^2+^ heme forms of cytochrome *c* is spectacularly shifted toward the reduced form Fe^2+^ upon retinoic acid and tyrosine activity in mitochondria decreases with increasing concentration of retinoic acid. These results may have far-reaching implications for cancer therapy. A quantitative approach to the measurement of receptor protein expression of tyrosine activity may improve specificity in selecting patients for triple-positive breast cancer targeted therapy. In the current study, we have used retinol binding protein (RBP) and STRA6 protein expression and cytochrome *c* in breast cancer cells as a model to explore the potential utility of a novel immunodetection technique, using Raman spectroscopy and Raman imaging, which can be quantitatively constructed by using cluster analysis. The paper provides experimental support for the theoretical hypothesis of how retinoic acid catalyzes resonance energy transfer reactions and controls the activation/inactivation cycle of protein kinase PKCδ. It has been proposed that reversible phosphorylation of cytochrome *c* mediated by cell signaling pathways is a primary regulatory mechanism that determines mitochondrial respiration, electron transport chain (ETC) flux, proton gradient ΔΨ_m_, ATP production, and ROS generation, linking oxidative phosphorylation to human cancer through a lack of energy, ROS production, cytochrome *c* release, and activation of apoptosis.

## Graphical abstract

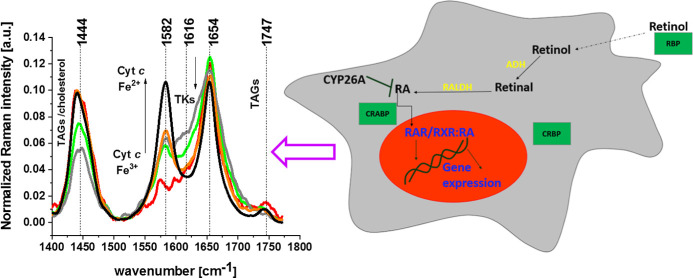

## 1 Introduction

To maintain biological complexity, living cells must communicate directly through contact or indirectly via chemical signaling. The signaling occurs via cellular receptors that are responsible for translating signals from outside the cell into signals within the cell. The receptors consist of an extracellular domain, a transmembrane segment, and an intracellular region. When signaling cascades are triggered by ligand-dependent (a ligand binds to a cell–surface receptor) or ligand-independent mechanisms (e.g., dimerization), the receptor’s intracellular domain changes in some way. The change in the intracellular domain induces a series of signaling events. These cascades of reactions can eventually lead to a change in the cell’s behavior or characteristics such as metabolic processes or transcription profile. Many of these receptors belong to a family of receptor tyrosine kinases (RTKs). The activated receptors phosphorylate multiple tyrosine (Y) residues at its C-terminus (Figure 1). Phosphorylation is an important covalent post-translational modification in cell signaling pathways. Protein phosphorylation is the reversible addition of a phosphate group to a protein or small molecule catalyzed by protein kinases. Upon activation, receptor tyrosine kinases (RTKs) facilitate cell-to-cell communication and regulate numerous intricate biological processes—such as cell growth, movement, differentiation, and metabolism—through their interactions with signaling pathways like PI3K/Akt, Raf/MEK/MAPK, JAK/STAT, Src, and PLCγ.^1,2^ (Figure 1).

**Figure 1:**
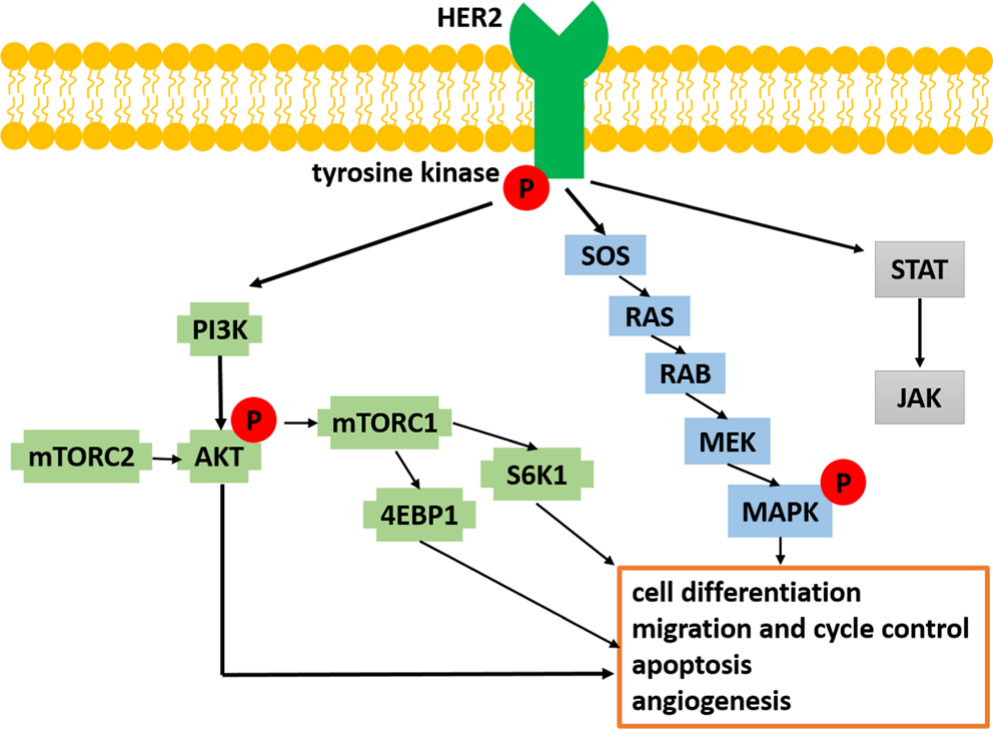
Main signaling pathways initiated by receptor tyrosine kinases (RTKs), symbol P indicates phosphorylation.

The study of global tyrosine phosphorylation is at the forefront of biomedical research because of its relation to human diseases, including cancers, by the abnormalities of receptor tyrosine kinases.

In normal cells, the activity of RTKs is closely regulated and properly balanced to keep cell growth under control. Inappropriate activation of RTK family receptors, overexpression of RTK receptors signal, or downregulation of RTK pathways provide important implications in cancer leading to uncontrolled growth and spread of tumor cells.^3^

Retinoic acid (RA) is an important signaling molecule that mediates intercellular communication (Figure 2). To understand the regulatory mechanisms, it is essential to obtain targeted and detailed experimental data on the relevant pathways. Nonetheless, practical approaches to experimental design remain underdeveloped. Here, we propose a Raman imaging-based experimental framework to identify regulatory mechanisms in cancers caused by retinoic acid.

**Figure 2:**
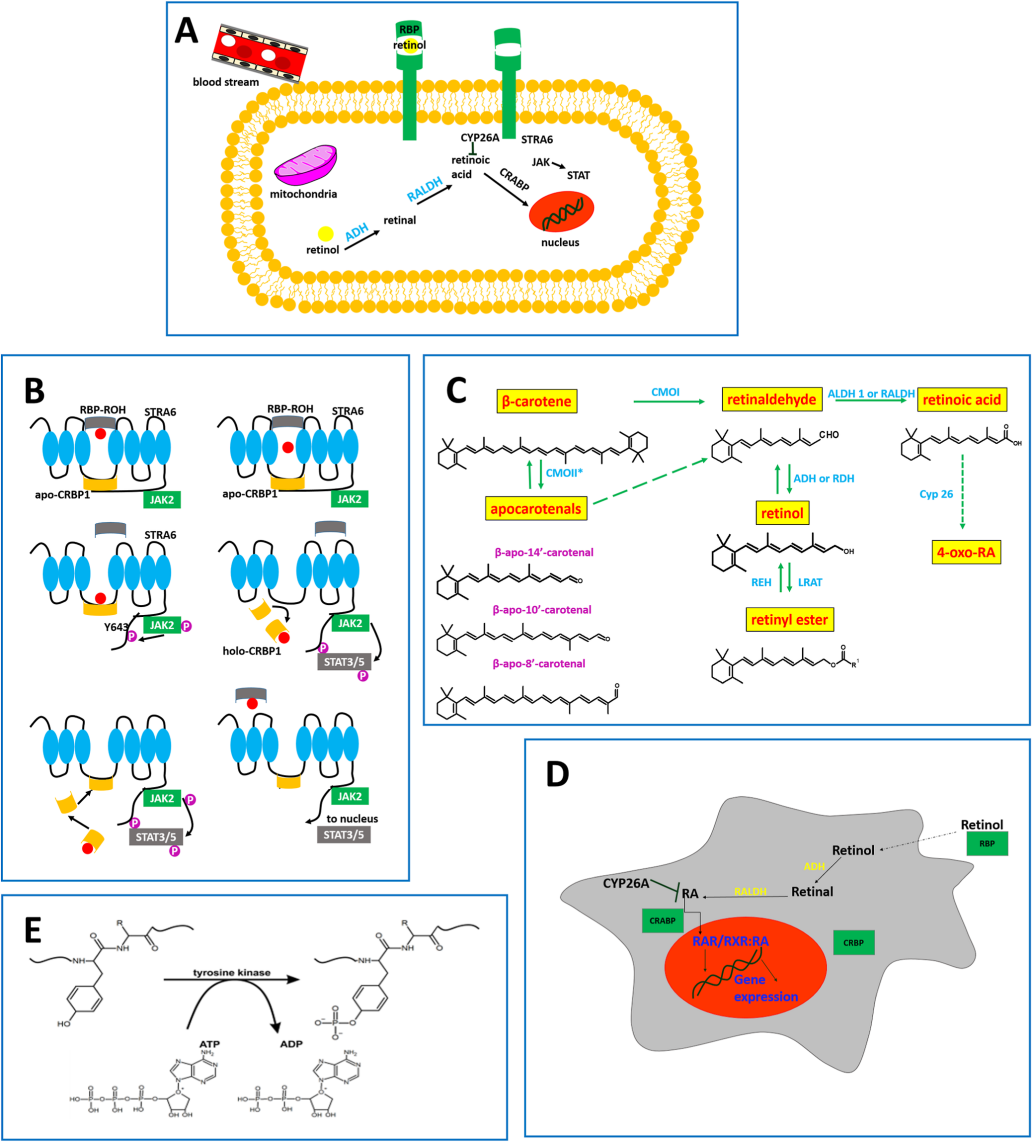
Retinoids and the retinoic acid (RA) signaling pathways in vertebrates. (A) Retinol, bound to retinol binding protein (RBP) and transthyretin (TTR), is transported via the bloodstream to target cells through the transmembrane receptor STRA6. (B) Mechanism of retinol transport inside cytosol to nucleus, mitochondria, and other organelles. The transfer of retinol to the interior of the cell occurs via CRBP1 protein attached to STRA6. This event activates the intracellular nonreceptor tyrosine kinase enzyme JAK2 and phosphorylation of STRA6 at Y643 (tyrosine). (C) Free retinol inside cytosol is transformed by cytosolic enzymes (ADHs/RolDHs, SDRs, ALDHs) into retinal, retinoic acid, retinyl esters and transported to the cell organelles. The retinoic acid is either degraded by the cytochrome P450 family (CYP26) to various inactive metabolites, or it is transported into the nucleus by cellular retinoic acid binding proteins (CRABPs). (D) In the nucleus, RA is bound to a heterodimer of retinoic acid receptor (RAR) and retinoid X receptor (RXR) and retinoic acid response elements (RAREs) leading to the initiation of gene transcription. (E) The effect of phosphorylation catalyzed by tyrosine kinase.

Retinoids are delivered to the human body via the diet from carotenoids or directly from food of animal origin, such as meat, fish, and dairy products. Retinol bound to retinol binding protein (RBP) is transported through the lymphatic or blood circulation and enters target cells through the transmembrane receptor STRA6 controlling the release of retinol from storage when it is needed.^4–7^ Retinoids and retinoic acid (RA) signaling pathways are presented in Figure 2.

To understand the role of retinoids in a cascade of metabolic and signaling events that are necessary to activate (or suppress) gene transcription in the nucleus, some clarification of interplay between metabolic functions and signaling is needed.^8,9^ We propose a novel method to monitor modifications induced in cells by retinoic acid at the molecular level via unique vibrational signatures of proteins with special attention to cytochrome *c*.

Raman spectroscopy and imaging are excellent tools that not only can provide a biochemical profile of cells but also can monitor accumulation and biocomposition alterations in specific organelles of cells as cancer progresses. The Raman method is capable of identifying metabolic mechanisms in breast cancer induced by retinoic acid.

Raman confocal microspectroscopy—a spectroscopic technique based on inelastic scattering of monochromatic light—does not require labeling of the molecules of interest and enables direct chemical imaging of specific organelles of cells: nucleus, mitochondria, endoplasmic reticulum, lipid droplets, cytoplasm, and membrane.^4,8,10–13^ We show that label-free Raman confocal microspectroscopy could also enable the clarification of the precise role of retinoids in the metabolism and signaling of cancer cells.

This work focuses on analyzing the role of retinoic acid in human breast cancer cell lines (MCF-7). MCF-7 cells possess an epithelial morphology and represent human triple-positive breast carcinoma. The growth of triple-positive breast cancer is driven by the activity of estrogen receptors, progesterone receptors, and human epidermal growth factor receptor 2 (HER2).^14^ MCF-7 is a noninvasive, low-aggression cell line typically characterized by limited metastatic potential.

In the current study, we studied human breast cancer cell lines as a model to explore the potential utility of a novel immunodetection technique by exploring Raman spectroscopy and Raman imaging combined with the cluster analysis described in^8,15,16^ to quantitatively measure retinoic acid expression. The present contribution provides a practical experimental Raman spectroscopic design approach that facilitates monitoring metabolic and signaling processes of molecular biology in carcinogenesis induced by retinoic acid.

## 2 Results

Retinoic acid (RA) is a crucial signaling molecule involved in intercellular communication, where tyrosine phosphorylation plays a major role as a post-translational modification (Figure 2E). The study initially concentrated on the Raman spectral changes occurring in tyrosine as a result of phosphorylation. To investigate tyrosine kinase activity, we first focused on analyzing the Raman spectra of both tyrosine and its phosphorylated form (Figure 3A).

**Figure 3:**
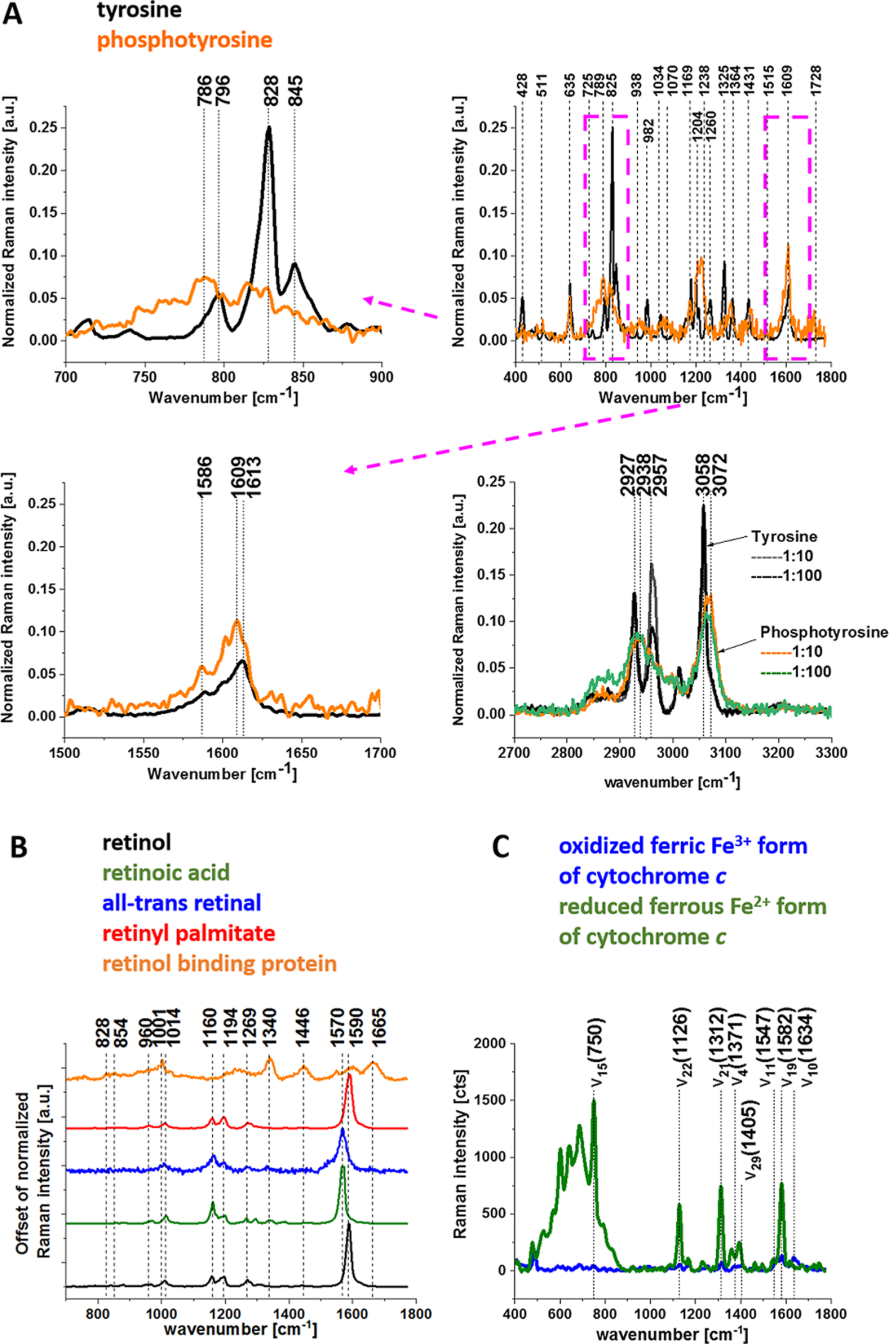
Raman spectra of tyrosine (black line) and phosphotyrosine (orange line) and Raman spectra of tyrosine and phosphotyrosine diluted in water solution (1:10, 1:100) (panel A); Raman spectra of retinoids and retinol binding protein in the fingerprint region (panel B); Raman spectra of isolated cytochrome *c* in solution (0.23 mM) excited at 532 nm dissolved in potassium phosphate buffer, pH 7.4, oxidized ferric Fe^3+^ (blue line), and reduced ferrous Fe^2+^ (green line) cytochrome *c*. Ferrous cytochrome *c* was prepared by adding a 10-fold excess of reductor ascorbic acid (panel C).

The phosphorylated forms of tyrosine can be monitored by using Raman scattering due to spectral changes in phosphorylated proteins arising from either phosphate stretching or amide vibrational modes.

Figure 3A shows the Raman spectra of tyrosine and phosphorylated tyrosine. A detailed inspection of Figure 3A presents that phosphorylation introduces noticeable Raman changes in tyrosine vibrations:

- phosphorylated tyrosine shows an additional Raman band at 1586 cm^–1^ near the main band at 1609 cm^–1^ that is attributed to the stretching mode of ring-O;^17^
- the band typical for phosphorylated tyrosine at 1609 cm^–1^ is shifted to 1613 cm^–1^ for tyrosine;
- a Fermi resonance between the first overtone of the aromatic out-of-plane ring bend and the aromatic ring breathing fundamental of tyrosine shows the characteristic doublet at 828 cm^–1^ and 845 cm^–1^ ^18^ and collapses into a single band with a significant intensity decrease after phosphorylation of tyrosine;
- Modes of the PO_4_^–^ phosphate group of phosphorylated tyrosine are visible at 1070 cm^–1^ and corresponds to the O–P–O symmetric stretching vibration,^18–23^

- upon phosphorylation, the Amide III band at 1260 cm^–1^ is shifted to 1238 cm–1;^19,20^

- the characteristic doublet (2927 cm^–1^, 2957 cm^–1^) of tyrosine collapses into a single band at 2938 cm^–1^ upon tyrosine phosphorylation with a significant intensity decrease;
- the band at 3058 cm^–1^ of tyrosine is shifted to 3072 cm^–1^ for phosphorylated tyrosine. While the different types of vibrations of tyrosine and phosphorylated tyrosine can be slightly different in position and shape in proteins due to their sensitivity to the microenvironment, their band positions vary only slightly by up to a few cm^–1^ in the Raman spectra of the proteins compared to the reference tyrosine and phosphorylated tyrosine.^24^

Having obtained the reference Raman fingerprint and the high-frequency region of tyrosine phosphorylation, we focused on the Raman spectral signatures of retinoids and the retinol binging protein.

Figure 3B shows the Raman spectra of retinoids and retinol binding protein. Figure 3B shows the Raman spectra of the following retinoids: retinol, retinoic acid, all-trans retinal, and retinyl palmitate, with the characteristic bands at 1570 cm^–1^ (retinoic acid, all-trans retinal) and 1590 cm^–1^ (retinol, retinyl palmitate). Retinol binding protein has a Raman band at 1665 cm^–1^, which is shifted with respect to typical Amide I at 1655 cm^–1^.

The typical protein kinase can add phosphates to 20 different proteins.^25^ One of the important proteins sensitive to phosphorylation is cytochrome *c*. It has been proposed that reversible phosphorylation of cytochrome *c* mediated by cell signaling pathways are primary regulatory mechanisms in higher organisms that determine mitochondrial respiration, electron transport chain (ETC) flux, proton gradient ΔΨ_m_, ATP production, and ROS generation, linking oxidative phosphorylation to human disease through a lack of energy, ROS production, cytochrome *c* release to cytoplasm, and activation of apoptosis.^26^

Figure 3C shows that the resonance Raman spectra of cytochrome *c* in resonance with *Q*_0_–*Q*_v_ (532 nm) electronic transitions are dominated by vibrational bands of the asymmetric A_2g_ modes, i.e., 1585 cm^–1^ (ν_19_), 1604 cm^–1^ (ν_38_), and the B_1g_ modes, i.e., 1638 cm^–1^ (ν_10_) and 1547 cm^–1^ (ν_11_). The vibrational assignments were taken from Spiro and Strekas^27^ and Hu et al.^28^ The other reports assigned the band at 1585 cm^–1^ to the ν_37_.^29^ The band at 1585 cm^–1^ is primarily due to the methine bridge vibrations via C_α_–C_m_ stretching and C_m_–H bending modes, respectively.^27,30,31^ The ν_15_ vibration at 750 cm^–1^ is associated with the deformation vibrations of the 16 membered inner ring of heme group, ν_4_ vibration at 1371 cm^–1^ involves breathing-like motion of pyrrole ring, ν_29_ vibration at 1405 cm^–1^ represents antisymmetric stretching C_a_-C_b_, the ν_19_ is mainly due to methine bridge stretching C_a_–C_m_ and C_a_-C_b_ vibrations mixed with C_m_–H bending mode with perpendicular displacement of the C_m_ atom to the plane of the heme group.^3^

Having obtained the reference Raman spectra of tyrosine phosphorylation, retinoids, retinol binding protein, and cytochrome *c*, we focused on the Raman spectral changes arising in specific organelles due to tyrosine phosphorylation and retinoids supplementation in cancerous breast in vitro cells.

Figure 4 presents the Raman images and Raman spectra for a standard MCF-7 control cell.

**Figure 4:**
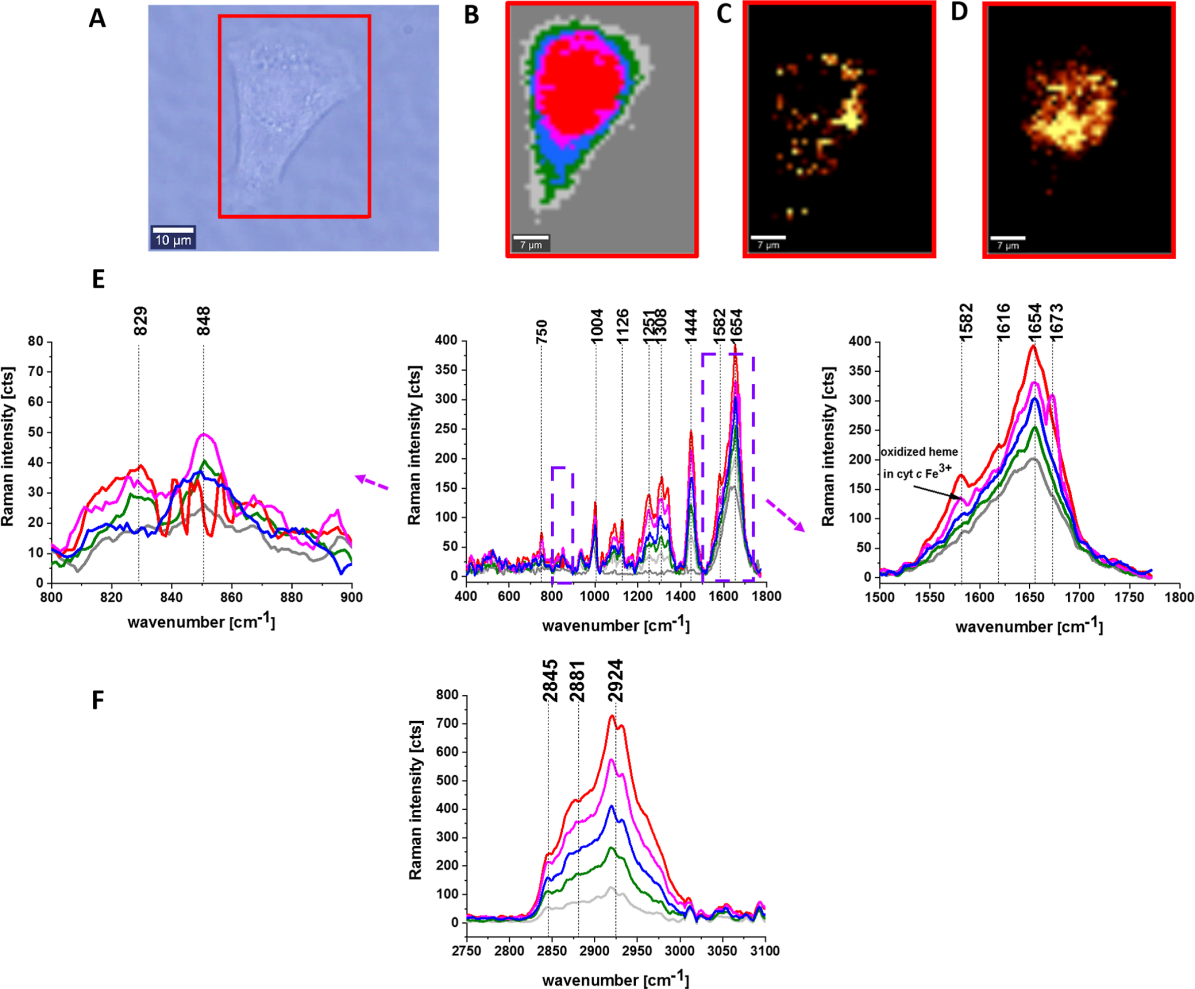
Raman imaging of a typical breast cancer cell MCF-7 (control). Microscopy images (A), Raman image (B) obtained from the cluster analysis (nucleus (red), endoplasmic reticulum (ER) (blue), lipid droplets (orange), cytoplasm (green), mitochondria (magenta), membrane (light gray)), fluorescence image of ER/lipid droplets (Oil Red O) (C), fluorescence image of nucleus (Hoechst 33342) (D), Raman spectra of the respective organelles (the same color as in Raman image) in the fingerprint region (E), and high-frequency region (F). The colors of spectra correspond to the colors of classes in the Raman maps.

Figure 4 shows that the Raman spectra of cancer breast cell of the MCF-7 line are dominated by cytochrome *c* due to Raman signal enhancement in resonance with *Q*_0_–*Q*_v_ (532 nm) electronic transitions. One can see that the vibrations of the heme group in cytochrome *c*: ν_15_ (750 cm^–1^), ν_22_ (1127 cm^–1^), ν_21_ (1310 cm^–1^), and ν_19_ (1582 cm^–1^) are the strongest bands in the breast cancer cell of MCF-7.

A detailed inspection of Raman spectra of specific organelles presented in Figure 4 shows the region of vibrations sensitive to phosphorylation by protein kinases on tyrosine vibrations (Figure 1B) and the region of cytochrome *c* (Figure 3C).

Detail inspection into Figure 4E shows that the characteristic doublet (829 cm^–1^, 848 cm^–1^) of tyrosine collapse into a single band as it happens upon tyrosine phosphorylation (Figure 3A). The vibrational features of tyrosine in the cancerous MCF-7 cells illustrate dynamic nature of phosphorylated proteins in a cell and suggest that the equilibrium between tyrosine and phosphorylated tyrosine is shifted toward phosphorylated tyrosine in all studied organelles of human breast cancer cells of MCF-7.

Figure 4C shows the Raman bands of cytochrome *c* at 1582 cm^–1^ and Amide I at 1654 cm^–1^ of a typical breast cancer cell (MCF-7). The equilibrium between ferric Fe^3+^ and ferrous Fe^2+^ forms in mitochondria and the other organelles and is dominated by the oxidized ferric cytochrome *c* Fe^3+^ in MCF-7 breast cancer cells. More details on this issue were recently published.^10,32^

Now, let us concentrate on the average Raman spectra normalized by the vector norm of breast cancer cells (MCF-7) for the cell organelles.

Figure 5 shows the average normalized Raman spectra of breast cancer cells (MCF-7) for the cell organelles: nucleus (red), endoplasmic reticulum (blue), lipid droplets (orange), cytoplasm (green), mitochondria (magenta), and membrane (light gray).

**Figure 5:**
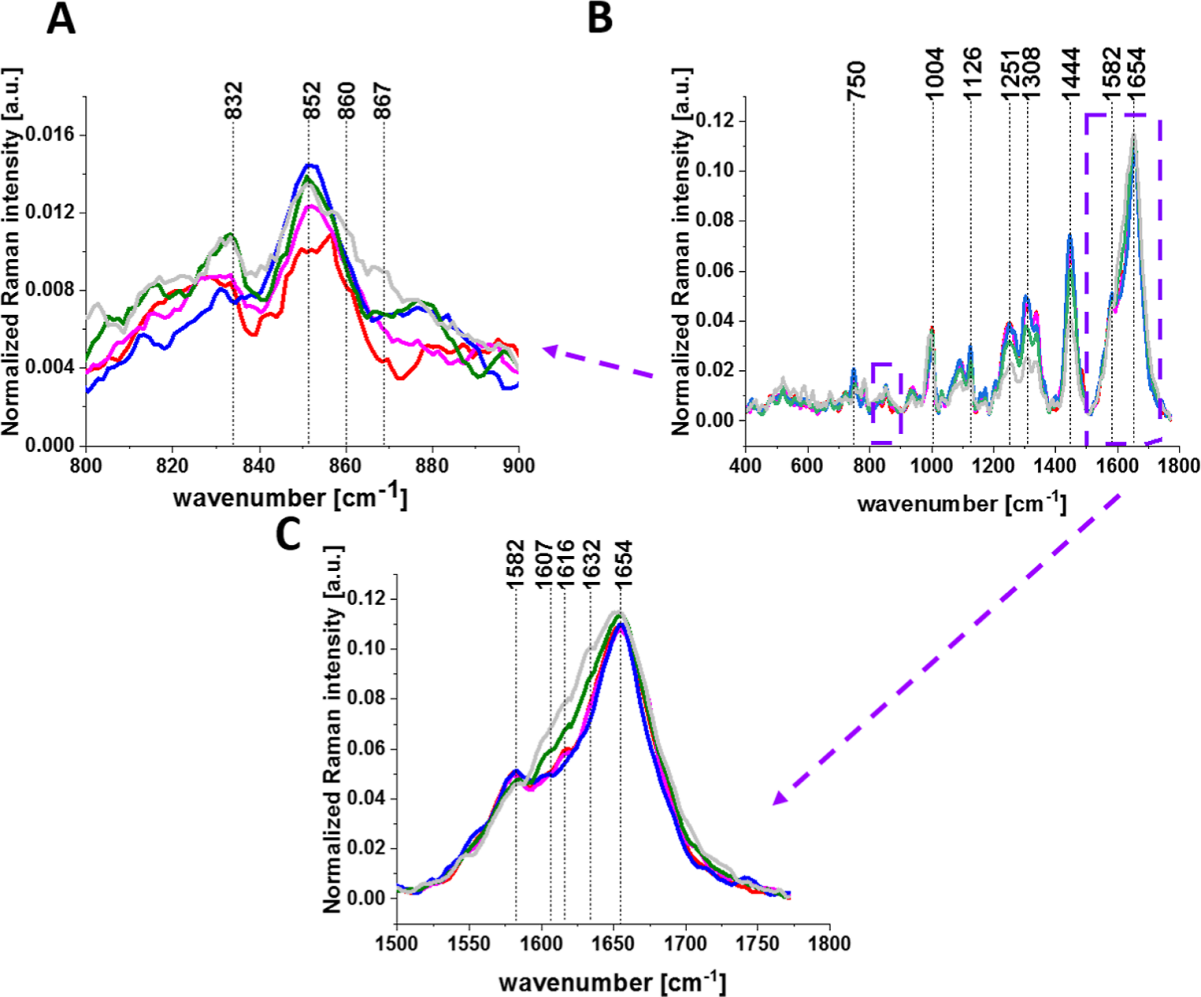
Average normalized Raman spectra of breast cancer cells (MCF-7) in the range 800–900 cm^–1^ (A), 400–1800 cm^–1^ (B), and 1500–1800 cm^–1^ (C) for the cell organelles nucleus (red), endoplasmic reticulum (blue), lipid droplets (orange) cytoplasm (green), mitochondria (magenta), and membrane (light gray).

Detailed inspection into Figure 5C shows that the most spectacular differences between the Raman signals for organelles occur in the region 1600–1655 cm^–1^ corresponding to tyrosine vibrations at 1607 cm^–1^ and 1616 cm^–1^ (Table 1). Indeed, the Raman signal for membrane and cytoplasm differs significantly from those in nucleus, mitochondria, lipid droplets, and endoplasmic reticulum. Figure 5C shows that overexpression of tyrosine vibrations is observed in the membrane and to a lesser extent in the cytoplasm.

**Table 1:** Vibrational Assignments of the Raman Bands Observed in MCF-7

MCF-7 triple-positive	vibrational assignments^9,28,33^
667	C–S stretching mode of cystine (collagen type I) T, G (DNA/RNA)
750	*c*, *c*1 and *b*-types of cytochrome ν _15_ deformation of inner ring of heme group
783	DNA: O–P–O, cytosine, uracil, thymin phosphatidylserine
826	tyrosine (Fermi resonance of ring fundamental and overtone)
848	tyrosine (Fermi resonance of ring fundamental and overtone)
860	tyrosine
867	monosaccharides (C–O–C) skeletal mode polysaccharides
936	skeletalC–C, a-helix
1004	phenylalanine
1084	C–C (lipid) symmetric phosphate stretching vibration of v_3_PO_4_
1126	c, c1 and b-types of cytochrome
1247	phosphorylated protein Amide III
1263	amide III
1442	cholesterol
1446	CH_2_ bending mode of proteins and lipids CH_2_ deformation
1456	CH_2_ stretching/CH_3_ asymmetric deformation overlapping asymmetric CH_3_ bending and CH_2_ scissoring (is associated with collagen and phospholipids)
1582	cytochrome *c* ν_19_ methine bridge vibrations via C_α_–C_m_ stretching and C_m_–H bending modes
1607	tyrosine
1616	tyrosine
1655	amide I of proteins, C=C of lipids
1745	triglycerides (fatty acids)
2846	CH_2_ symmetric stretch of lipids
2884	CH_2_ asymmetric stretch of lipids and proteins
2929	symmetric CH_3_ stretch due primarily to protein
2964	v_as_ CH_3_, lipids, fatty cholesterol, and cholesterol ester
3058	tyrosine
3072	phosphorylated tyrosine

The membrane of cells contains many cellular receptors that are responsible for translating signals from outside the cell into signals within the cell. The receptors consist of an extracellular domain, a transmembrane segment, and an intracellular region. Many of these receptors such as HER2, EGFR, or STRA6 (Figures 1 and 2) belong to a family of receptor tyrosine kinases (RTKs). The activated receptors phosphorylate multiple tyrosine (Y) residues at their C-termini (Figures 1 and 2B). The spectral region 1600–1655 cm^–1^ in the cell membrane presented in Figure 5C represents HER2, EGFR, and STRA6 receptors, but only STRA6 can be modified by retinoic acid. STRA6 is a transmembrane protein that serves as a receptor for retinol binding proteins (RBPs), which are key carriers of retinol.^34^ Therefore, it is easy to separate STRA6 from the other RTKs (Figure 2).

In this article, we will concentrate on the retinol binding proteins (RBPs) on the surface of the membrane and the transmembrane receptor STRA6 involved in transport of retinol to the interior of the cell. The retinol is transported through the CRBP1 protein, which is attached to the CRBP binding loop on STRA6 (Figure 2B). This process triggers the activation of the intracellular nonreceptor tyrosine kinase JAK2, which phosphorylates STRA6 at Y643 (tyrosine).

To understand the role of retinoids in a cascade of metabolic and signaling events that are necessary to activate (or suppress) gene transcription in the nucleus, some clarification of interplay between metabolic functions and signaling is needed. To improve our knowledge on the role of retinoids in these processes, we recorded the Raman spectra and images of cells receiving redox stimuli by retinoic acid (RA) in in vitro cell cultures. For these purposes, we incubated human breast cancer cells MCF-7 with RA at concentrations of 1, 10, and 50 μM for 24 and 48 h of incubation.

Free retinol inside the cytosol is transformed by cytosolic enzymes into retinal, retinoic acid, and retinyl esters that are transported to the cell organelles: nucleus, mitochondria, ER, and lipid droplets as retinol, retinal, retinyl esters, and retinoic acid. We will study accumulation of retinoic acid and redox status of cytochrome *c* in specific organelles to clarify interplay between metabolic and signaling functions of retinoic acid in cancer cells. First, let us concentrate on the retinoic acid accumulation in mitochondria.

### 2.1 Mitochondria

Figure 6 shows the normalized average Raman spectra in the range 400–1800 cm^–1^ for the mitochondria of human breast cancer cells (MCF-7) without (control) and incubated with retinoic acid at concentrations of 1, 10, and 50 μM for 24 and 48 h.

**Figure 6:**
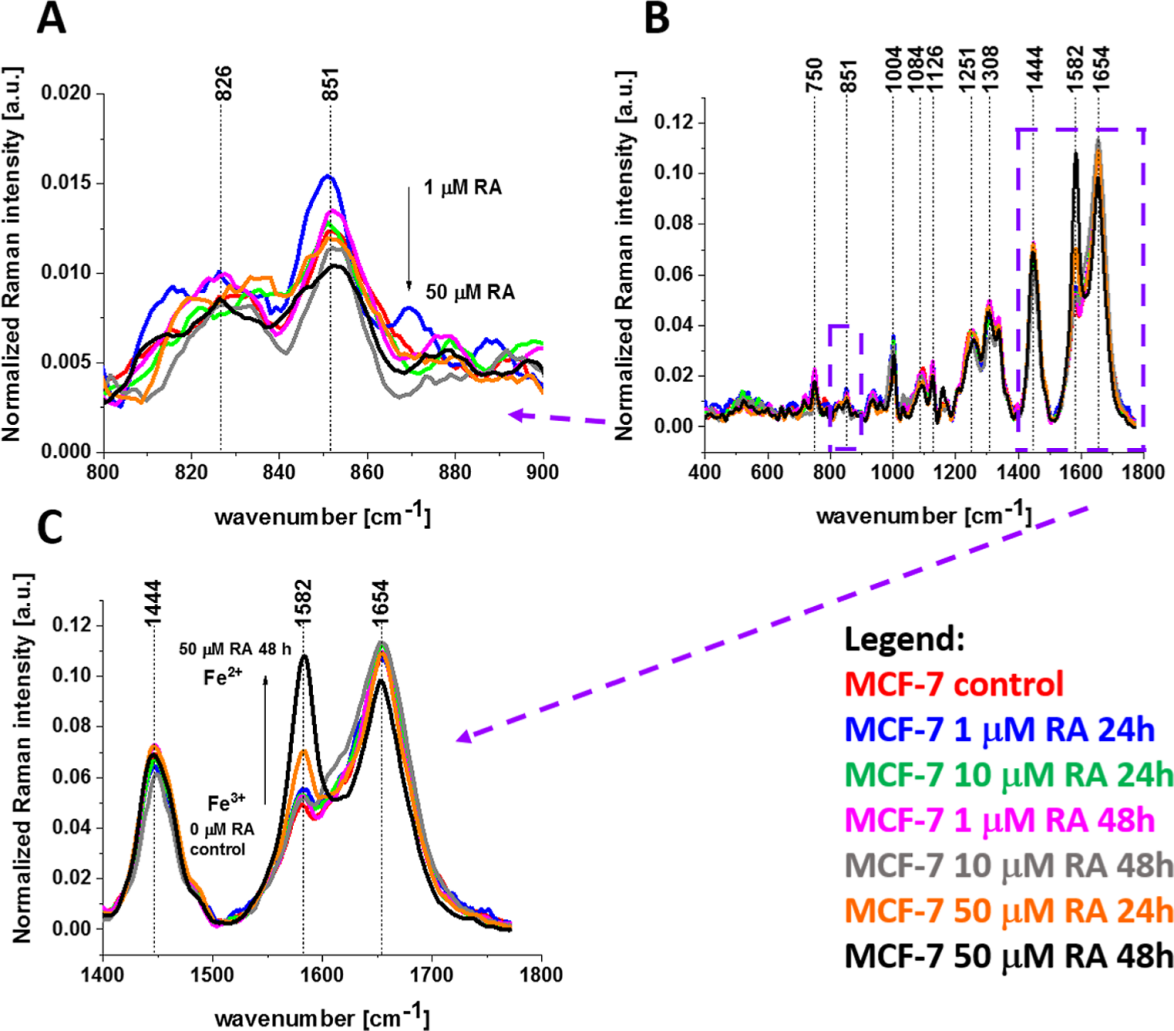
Normalized average Raman spectra in the range 800–900 cm^–1^ (A), 400–1800 cm^–1^ (B), and 1400–1800 cm^–1^ (C) for the mitochondria of human breast cancer cells (MCF-7) without (control) and incubated with retinoic acid at a concentration of 1, 10, and 50 μM for 24 and 48 h.

The most striking effect of retinoic acid is a dramatic increase in the Raman signal of the ν_19_ band at 1582 cm^–1^ of cytochrome *c* observed upon incubation with retinoic acid. This enhancement of the Raman signal provides clear evidence that retinoic acid affects the redox status of the heme group of cytochrome *c* shifting the Fe^3+^/Fe^2+^ equilibrium from the oxidized iron ion Fe^3+^ to the reduced Fe^2+^ as we showed in Figure 6C. This conclusion comes from the fact that the Raman bands of the reduced form of cytochrome *c* have much higher intensities than those of the oxidized form, as shown in Figure 3C.

To understand how retinoic acid affects the redox status of the heme group of cytochrome *c* by shifting the Fe^3+^/Fe^2+^ equilibrium from the oxidized iron ion Fe^3+^ to the reduced Fe^2+^, we determined the effect of retinoic acid on the redox status of cytochrome *c* in mitochondria. We compared the Raman spectra of the control cells (without retinoic acid) with those of the cells receiving redox stimuli by retinoic acid in in vitro cell cultures. The results are presented in Figure 7.

**Figure 7:**
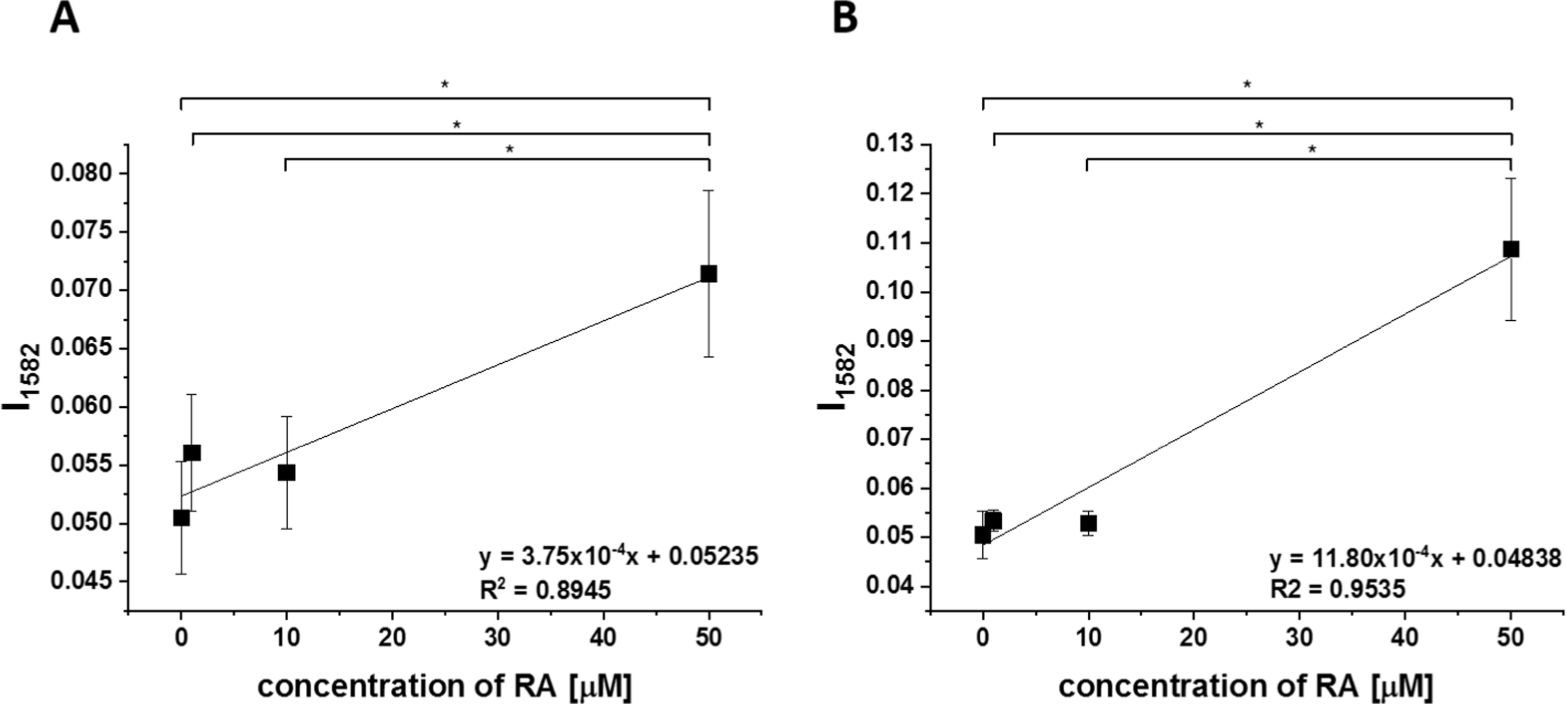
Average normalized Raman band ν_19_ intensity of cytochrome *c* in mitochondria of the MCF-7 cell at 1582 cm^–1^ as a function of retinoic acid concentration after 24h (A) and 48 h (B) of incubation, asterisk * shows statistical significance, *p*-value ≤0.05 according to ANOVA.

Figure 7 shows the Raman band ν_19_ intensity at 1582 cm^–1^ in mitochondria of MCF-7 cell as a function of retinoic acid concentration. It is evident that at low retinoic acid concentrations, up to 10 μM, the intensity of the Raman signal in mitochondria does not change significantly, indicating that cytochrome *c* still remains in the oxidized state with the Fe^3+^ iron ion of the heme group. For higher concentrations, retinoic acid dramatically changes the redox status of cytochrome *c* to the reduced Fe^2+^. The physiological concentrations of retinoic acid in the blood are around 3–140 nM.^35^ The results suggest that at a concentration of 50 μM, retinoids could overwhelm normal metabolism and provoke toxicity.

Kéri et al. reported that treating SW620, HT29, and COLO205 cells with 10 μM all-trans retinoic acid reduced both their proliferation rates and tyrosine kinase activity, whereas SW480 cells were resistant to the treatment.^36,37^

Retinoic acid is an important signaling molecule mediating intercellular communication, in which tyrosine phosphorylation is one of the most important steps in post-translational modifications in cells. We analyzed the vibrations of tyrosine in mitochondria upon incubation with retinoic acid.

Figure 6A shows that retinoic acid decreases the Raman signal at 851 cm^–1^ in mitochondria, indicating that it decreases the tyrosine kinase activity in cancer breast cells of MCF-7. The same conclusion is obtained from other tyrosine vibrations.

Figure 8 shows that retinoic acid decreases the Raman signals at 3058 cm^–1^ (tyrosine) and 3072 cm^–1^ (phosphorylated tyrosine) in mitochondria, indicating that retinoic acid reduces the tyrosine kinase activity in cancerous breast cells of MCF-7.

**Figure 8:**
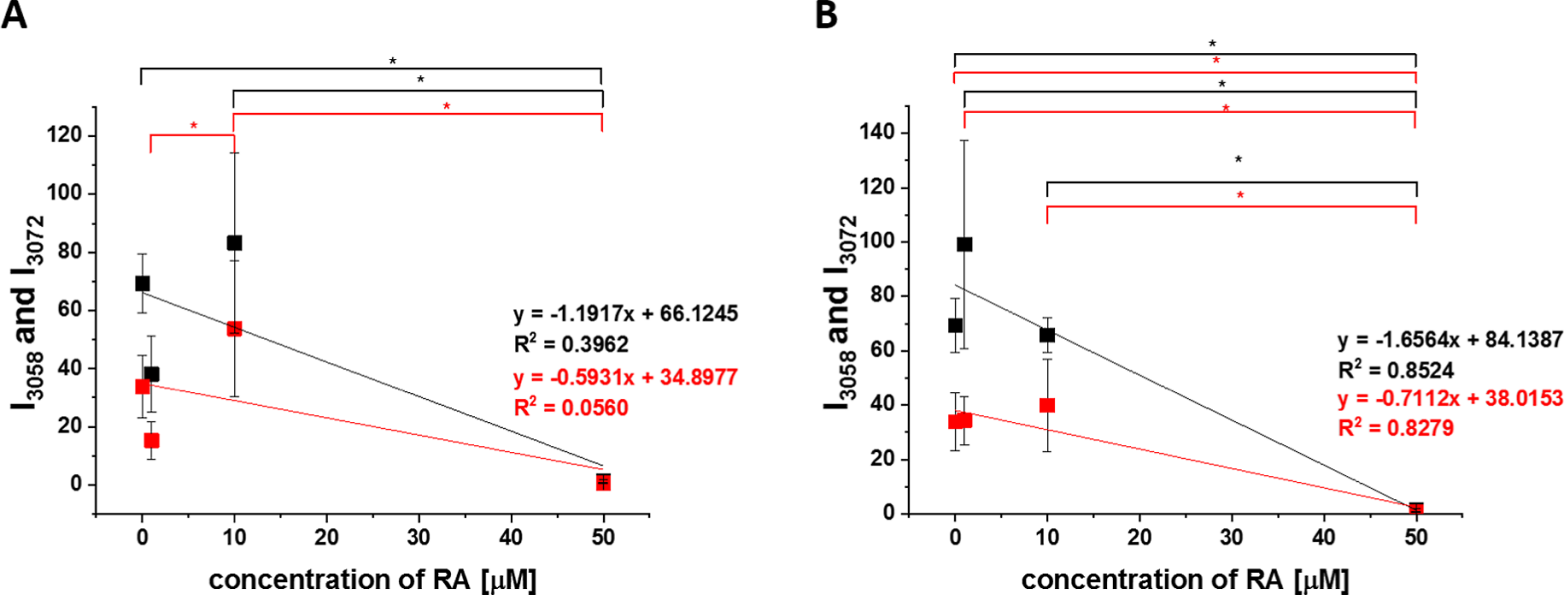
Raman signals at 3058 cm^–1^ (tyrosine; black line) and 3072 cm^–1^ (phosphorylated tyrosine; red line) in mitochondria as a function of retinoic acid concentration, incubation for 24 h (A) and 48 h (B), asterisk * shows statistical significance, *p*-value ≤0.05 according to ANOVA.

We showed in Figure 5C that the most spectacular Raman features of cellular receptors of MCF-7 cancer cells occur in the membrane in the region 1600–1655 cm^–1^ corresponding to tyrosine vibrations at 1607 cm^–1^ and 1616 cm^–1^ (Table 1). It is interesting to see whether retinoic acid modifies the cellular receptors. Figure 9 compares the Raman signals of the membrane and mitochondria in a typical human breast cancer cell MCF-7 incubated with 50 μM retinoic acid.

**Figure 9:**
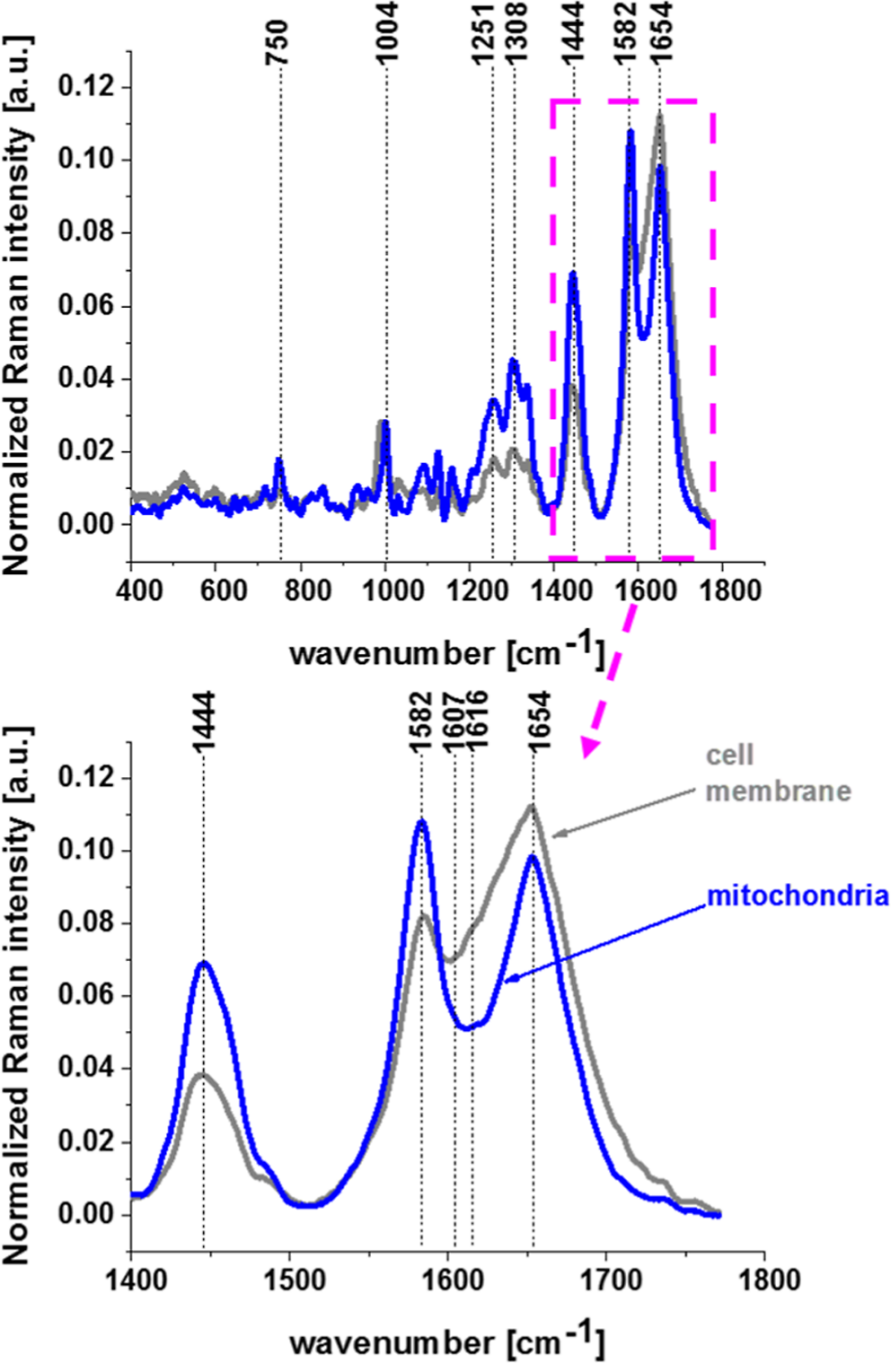
Raman signals of membrane and mitochondria in a typical human breast cancer cell MCF-7 incubated with 50 μM of retinoic acid, cell membrane (gray line), and mitochondria (blue line).

Figure 9 shows evidently that cellular receptors on the surface of cancer cells incubated with 50 μM of retinoic acid in the spectral region 1600–1655 cm^–1^ corresponding to tyrosine vibrations at 1607 cm^–1^ and 1616 cm^–1^ (Table 1) give spectacularly higher Raman signals in the membrane than in mitochondria. This region represents the transmembrane cell protein STRA6.

To understand how retinoic acid affects tyrosine kinase activity and the redox status of the heme group of cytochrome *c*, we determined the effect of retinoic acid in other specific organelles: endoplasmic reticulum, lipid droplets, cytoplasm, and membrane.

First, let us concentrate on the transfer of retinoic acid into endoplasmic reticulum (ER), where LRAT (lecithin: retinol acyltransferase) at the membrane of ER catalyzes retinol esterification.^38^

### 2.2 Endoplasmic Reticulum and Lipid Droplets

Figure 10 shows the normalized average Raman spectra in the ranges 400–1800 cm^–1^ and 2750–3100 cm^–1^ for the endoplasmic reticulum (panel A) and lipid droplets (panel B) of human breast cancer cells (MCF-7) without (control) and incubated with retinoic acid as a function of retinoic acid concentration.

**Figure 10:**
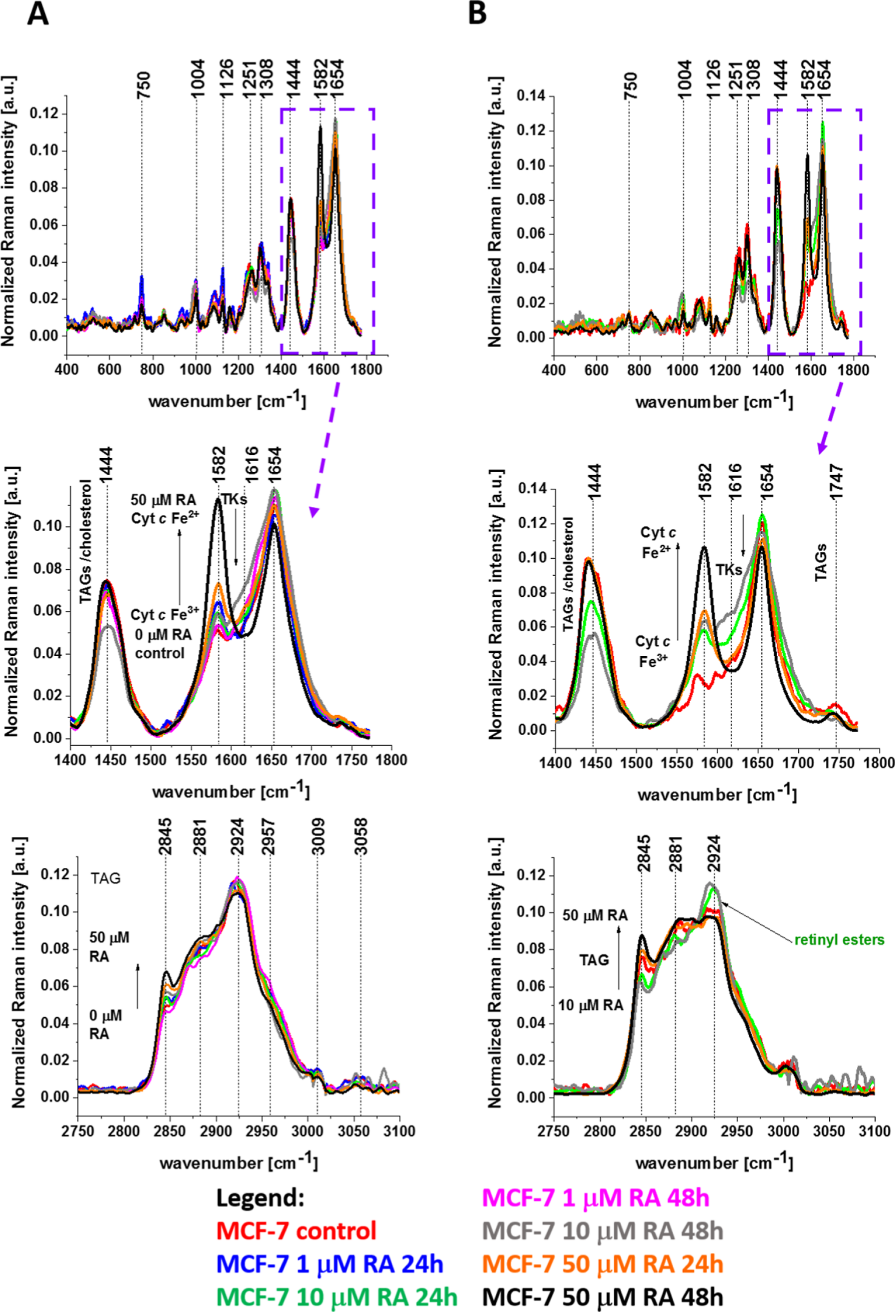
Normalized average Raman spectra in the range 400–1800 cm^–1^ and 2750–3100 cm^–1^ for the endoplasmic reticulum (panel A) and lipid droplets (panel B) of human breast cancer cells (MCF-7) without (control) and incubated with retinoic acid at a concentration of 1, 10, and 50 μM for 24 and 48 h.

Comparison between Figure 3B for retinyl ester (retinyl palmitate) and the Raman spectra in Figure 10A in the high-frequency region of 2750–3100 cm^–1^ clearly indicates that ER contains retinyl esters, supporting the mechanism of LRAT esterification in breast cancer cells of MCF-7.

This conclusion is more universal because we reported recently that the retinyl ester vibrations were identified in lipid structures of ER as a profile II of NHA normal astrocyte cells in human brain cells.^4^ Figure 10A shows that ERs contain cytochrome *c* demonstrated by a strong signal at 1582 cm^–1^. The Raman signal at 1582 cm^–1^ provides clear evidence that retinoic acid affects the redox status of the heme group of cytochrome *c* in ER, like in mitochondria, shifting the Fe^3+^/Fe^2+^ equilibrium from the oxidized iron ion Fe^3+^ to the reduced Fe^2+^ with an increase of retinoic acid concentration (Figure 6C).

To gain deeper insight into metabolic processes of lipid reprogramming in cancer cells, we studied how retinoic acid affects lipid droplets. Recently, we have showed the lipid droplets in brain cancer cells are predominantly filled with triglycerides (TAGs) and are involved in energy storage.^4^ Figure 10B shows normalized average Raman spectra in the range 400–1800 cm^–1^ and 2750–3100 cm^–1^ for lipid droplets of human breast cancer cells (MCF-7) without (control) and incubated with retinoic acid as a function of retinoic acid concentration. Figure 10B shows that the retinoic acid induces the increase in the concentration of triglycerides (TAGs) illustrated by the vibration at 2845 cm^–1^. The lipid droplets in human breast cancer cells are predominantly filled with TAGs at 50 μM concentration of retinoic acid that are involved in energy storage. At a lower concentration of retinoic acid (10 μM), the lipid droplets are filled mainly with retinyl esters/retinoic acid and are likely involved in signaling, especially JAK2/STAT6 pathway signaling. Figure 10B shows that lipid droplets contain cytochrome *c*, which is illustrated by a strong Raman signal at 1582 cm^–1^. The Raman signal at 1582 cm^–1^ provides clear evidence that retinoic acid affects the redox status of the heme group of cytochrome *c* shifting the Fe^3+^/Fe^2+^ equilibrium from the oxidized iron ion Fe^3+^ to the reduced Fe^2+^ with an increase of retinoic acid concentration like in ER and in mitochondria (Figure 9).

### 2.3 Nucleus

Retinoic acid signaling is regulated by proteins of the cytochrome P450 family, chiefly CYP26, which oxidize RA to various inactive metabolites, including 4-oxo retinoic acid, which are believed to be transcriptionally inactive, or it is transported into the nucleus by cellular retinoic acid binding proteins (CRABPs). Inside the nucleus, RA binds to a heterodimer of the retinoic acid receptor (RAR) and retinoid X receptor (RXR). Gene transcription is initiated when the RAR/RXR heterodimer binds to RAREs within the regulatory region of retinoic acid.^4–13^

Figure 11 shows normalized average Raman spectra in the range 400–1800 cm^–1^ and 2750–3100 cm^–1^ for the nucleus of human breast cancer cells (MCF-7) without (control) and incubated with retinoic acid at a concentration of 1, 10, and 50 μM for 24 and 48 h.

**Figure 11:**
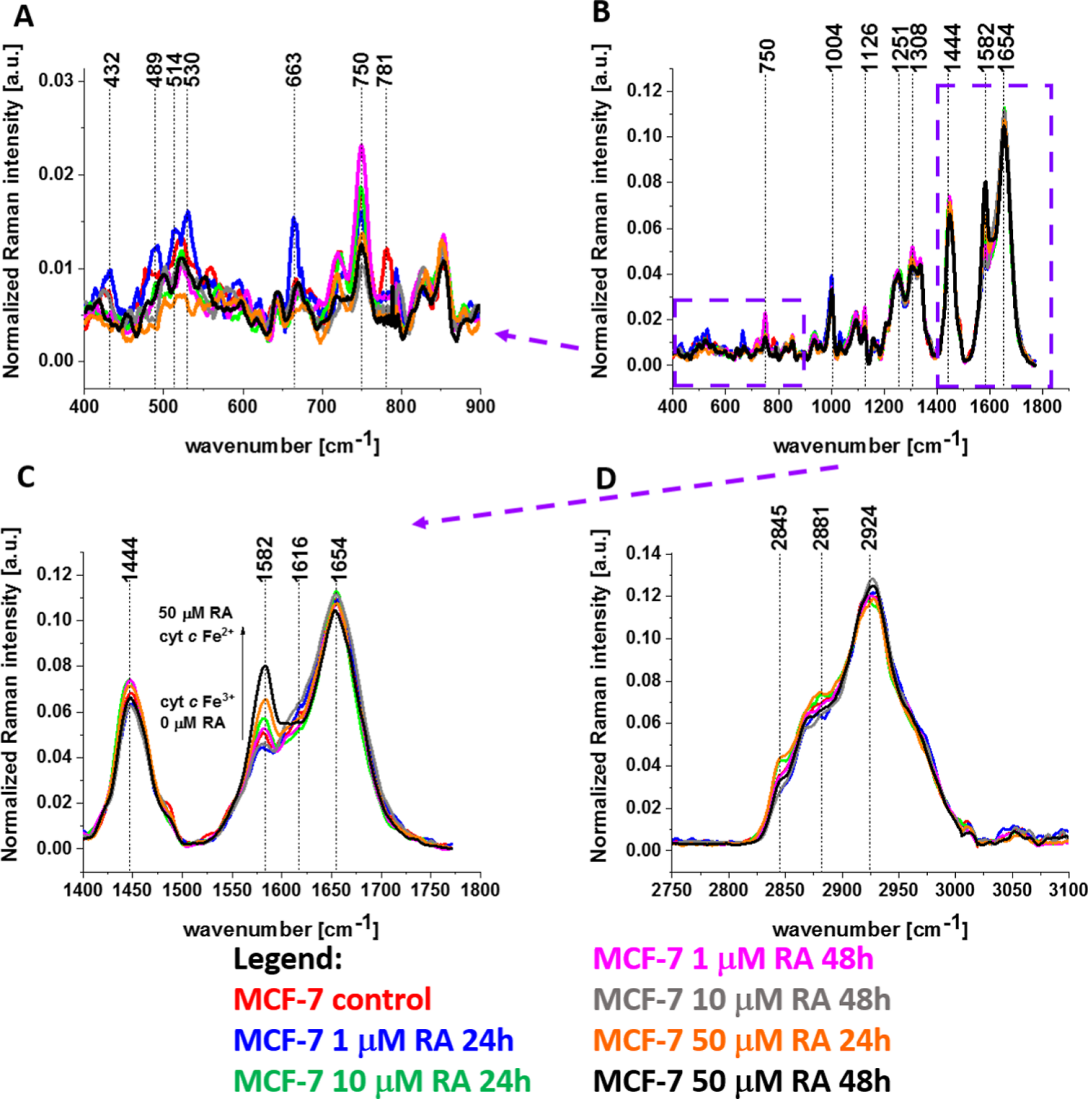
Normalized average Raman spectra in the range 400–900 cm^–1^ (A), 400–1800 cm^–1^ (B), 1400–1800 cm^–1^ (C), and 2750–3100 cm^–1^ (D) for nucleus of a human breast cancer cells (MCF-7) without (control) and incubated with retinoic acid at a concentration of 1, 10, and 50 μM for 24 and 48 h.

A detailed inspection of Figure 11 shows that the Raman nucleus spectra exhibit changes upon incubation with retinoic acid. The changes are particularly visible in the region of ring breathing modes in the DNA bases-tyrosine-G backbone in RNA, uracil-based ring breathing mode, cytosine/uracil ring breathing (nucleotide), DNA, thymine, cytosine, uracil, RNA, U, T, and C (ring breathing modes in the DNA/RNA bases)^35^ (Figure 11A, Table 1).

The identification of RAR, RXR, RAREs, and associated coactivator complexes leading to the initiation of gene transcription or chromatin remodeling and DNA damage is rather difficult, and more advances in Raman methodology will be needed in the future to identify these alterations. This topic is beyond the scope of this paper.

Figure 11C shows that cytochrome *c* is also localized in the nucleus, not only in mitochondria, ER, and lipid droplets. This finding sheds new light on a novel role for cytochrome *c* in inducing nuclear apoptosis by the remodeling of chromatin. Our results support the suggestions that nuclear cytochrome *c* targets a variety of well-known histone chaperones involved in chromatin remodeling and DNA damage response.^39–42^

### 2.4 Cytoplasm and Cell Membrane

Figure 12 shows normalized average Raman in the ranges of 400–1800 cm^–1^ and 2750–3100 cm^–1^ for the cytoplasm and cell membrane of human breast cancer cells (MCF-7) without (control) and incubated with retinoic acid at concentrations of 1, 10, and 50 μM for 24 and 48 h.

**Figure 12:**
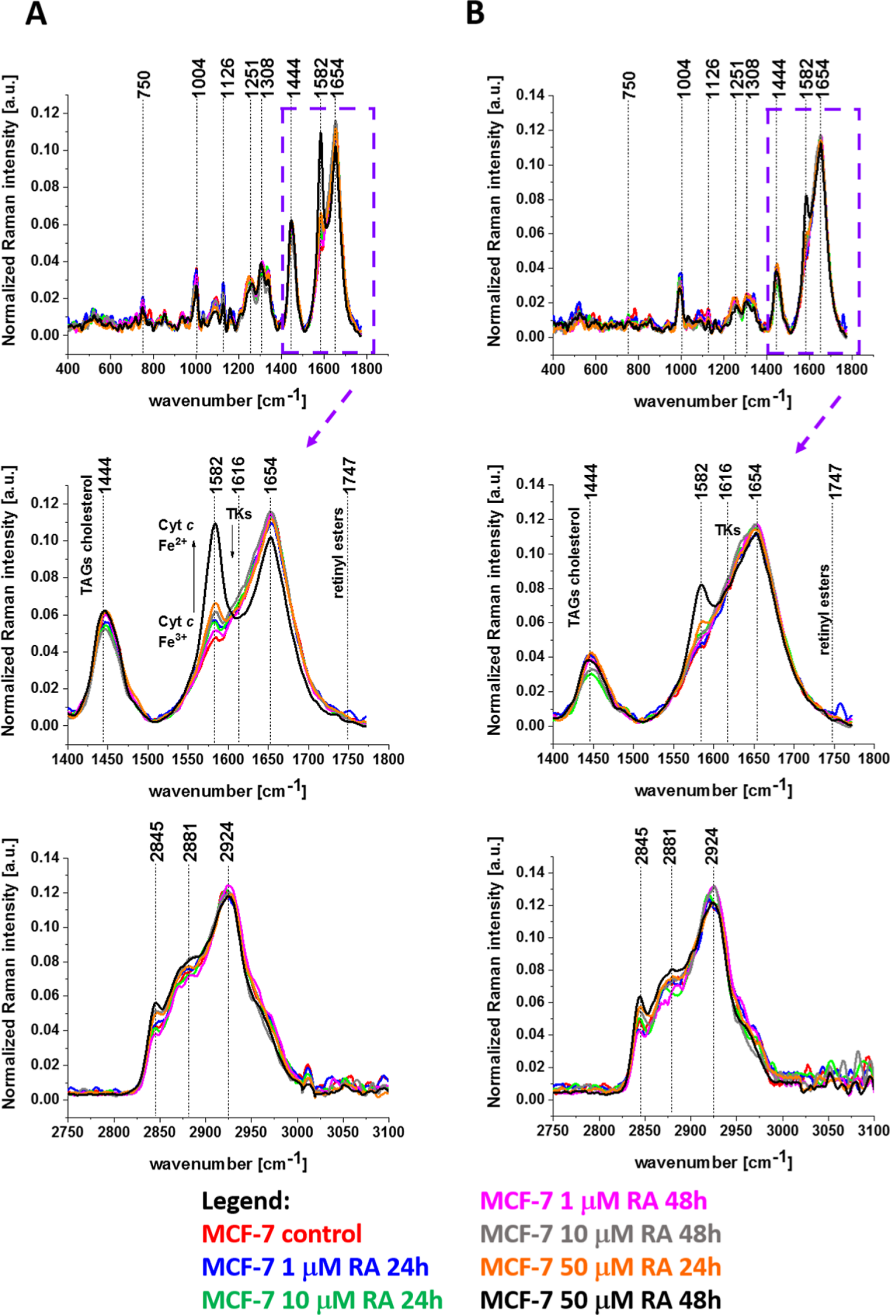
Normalized average Raman spectra in the range 400–1800 cm^–1^ and 2750–3100 cm^–1^ for cytoplasm (panel A) and cell membrane (panel B) of a human breast cancer cells (MCF-7) without (control) and incubated with retinoic acid at a concentration of 1, 10, and 50 μM for 24 and 48 h.

Free retinol inside cytosol is transformed by cytosolic enzymes into retinal, retinoic acid, and retinyl esters and transported to the cell organelles. Apoptosis induced by mitochondrial cytochrome *c* occurs in cytoplasm. Therefore, it is very important to monitor processes in the cytoplasm upon incubation with retinoic acid.

The Raman signal at 1582 cm^–1^ provides clear evidence that retinoic acid affects the redox status of the heme group of cytochrome *c* in cytoplasm shifting the Fe^3+^/Fe^2+^ equilibrium from the oxidized iron ion Fe^3+^ to the reduced Fe^2+^ with an increase of retinoic acid concentration like in mitochondria, ER, lipid droplets, and nucleus.

As we are interested in mechanisms of retinol transport to the interior of the cell, we concentrated on processes occurring in cell membranes. The surface of the membrane of cells contains many cellular receptors that are responsible for translating different signals from outside the cell into signals within the cell (Figure 1), but in this paper, we focus only on the retinol binding proteins (RBPs) receptors discussed in Figure 2.

Figure 12B shows normalized average Raman in the ranges 400–1800 cm^–1^ and 2750–3100 cm^–1^ for the cell membrane of human breast cancer cells (MCF-7) for the control cells (without retinoic acid) and incubated with retinoic acid at a concentration of 1, 10, and 50 μM for 24 and 48 h.

Our results, presented in Figure 12B shows that the tyrosine activity in the TKs region does not depend on retinoic acid concentration. It indicates that the number RBP receptors does not depend on retinoic acid concentration and characterizes the surface of the cell itself.

### 2.5 Comparison of Different Organelles in Cancer Cells

To summarize the effect of retinoic acid on different organelles, we compared the tyrosine activity TKs at 1616 cm^–1^ for different organelles. Figure 13 shows the tyrosine activity at 1616 cm^–1^ for different organelles in MCF-7 cells supplemented with 50 μM RA after 24 and 48 h of incubation.

**Figure 13:**
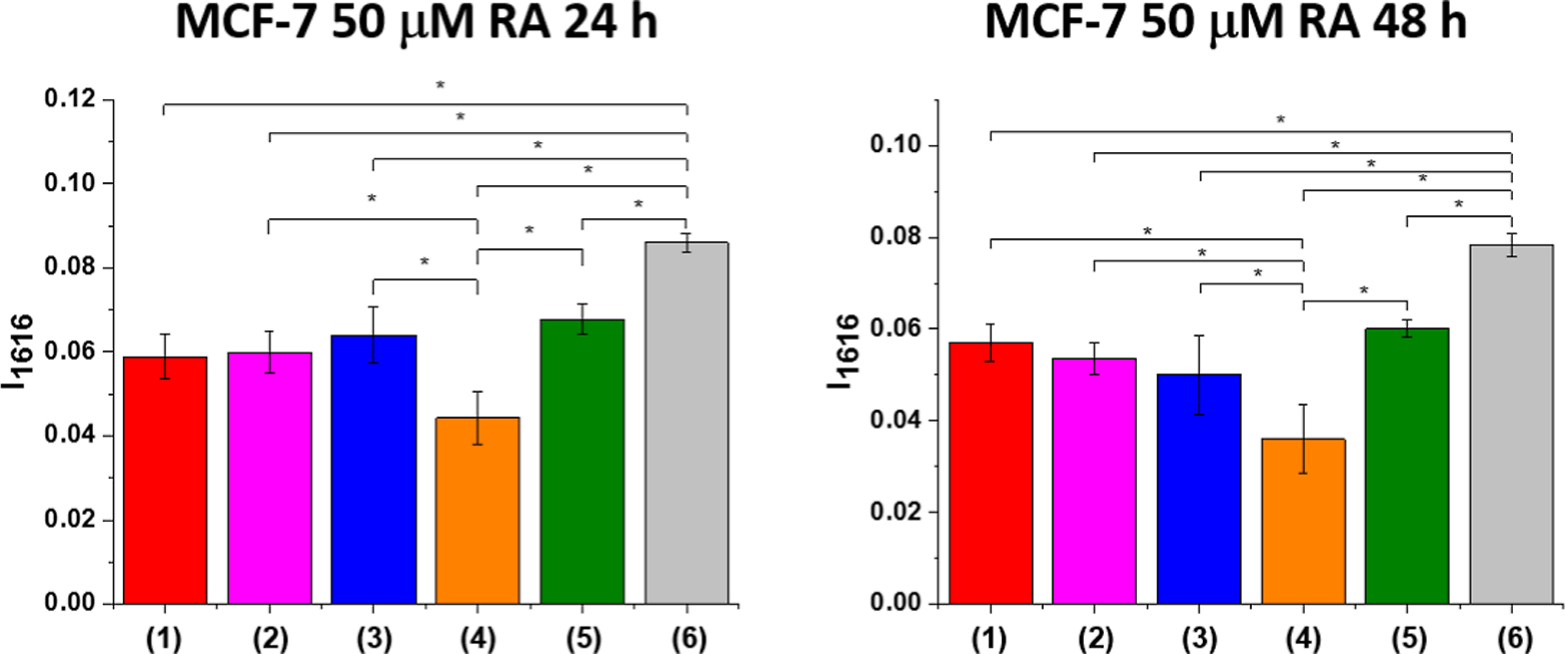
Tyrosine activity (TKs) at 1616 cm^–1^ in MCF-7 supplemented with RA after 24 and 48 h incubation; (1) nucleus (red bar), (2) mitochondria (magenta bar), (3) endoplasmic reticulum (orange bar), (4) lipid droplets (blue bar), (5) cytoplasm (green bar), and (6) cell membrane (gray bar), asterisk * shows statistical significance, p-value ≤ 0.05 according to ANOVA.

One can see that the TKs activity after 24 and 48 h of incubation with retinoic acid is the highest in membrane due to the RBP/STRA6 receptors on its surface and in cytoplasm followed by nucleus, mitochondria, ER, and lipid droplets.

## 3 Discussion

Over the past decade, evidence has emerged that cytochrome *c* plays a crucial global role in normal and cancer cell metabolism.^39–41,43–51^ In the paper, we showed functional diversity of cytochrome *c* in the mitochondria, ER, lipid droplets, cytoplasm, and nucleus upon incubation with retinoic acid.

The most important issue of this paper is understanding the role of retinoic acid in human breast triple-positive cancers. We showed that in all studied organelles, retinoic acid shifts the equilibrium from the oxidized Fe^3+^ form to the reduced Fe^2+^ form of cytochrome *c*. This shift has very serious implications on ETC flux, ΔΨ_m_, ATP production, and ROS generation, as will be discussed below. The mechanism of alteration of the redox status of cytochrome *c* is still unknown. Recent reports propose the model to explain this mechanism.^10^

In the view of the recent research studies^8,10,52,53^ and the results presented in the paper, we propose a model that couples the metabolic and signaling functions of retinoic acid in cancer cells.

Practical experimental design approaches to monitor the metabolic and signaling functions of retinoic acid in cancers are still in their infancy. To understand the underlying regulatory mechanisms, it is essential to obtain pathway-specific and informative experimental data for metabolic changes within each organelle. In this article, we proposed an experimental framework based on Raman imaging for identification of regulatory mechanisms in cancers induced by retinoic acid.

We showed that the tyrosine kinase activity on the cell surface and phosphorylation in transmembrane proteins including STRA6 receptors can be monitored by the vibrations of TKs receptors. The mitochondrial activity can be monitored by vibrations of cytochrome *c* and alterations in redox status of cytochrome *c*.

Figures 6–9 illustrate the fate of retinoic acid in mitochondria. The shift from the oxidized Fe^3+^ form to the reduced Fe^2+^ form of cytochrome *c* has very serious implications. The reduced form of cytochrome *c* generated by retinoic acid in mitochondria cannot, at the outset, activate the apoptosome or induce apoptosis or cytokine signaling in the cytoplasm. In essence, it does not serve as a universal DAMP damage-associated molecular pattern that warns the immune system of danger in various cells or tissues, as it blocks the activation of conventional mitochondrial signaling protein pathways. Moreover, an excess of reduced cytochrome *c* in the mitochondrial ETC suggests a risk of mitochondrial dysfunction, including impaired electron flow between complexes III and IV, increased ROS production, disrupted ATP synthase activation by the electrochemical gradient, and reduced oxidative phosphorylation efficiency. All of these processes can be explained by the traditional channel of the ETC presented in Figure 14 and indicated as channel I. A longstanding point of debate concerns which specific step in the ETC governs overall electron flow and acts as the rate-limiting bottleneck. Maintaining tightly controlled activity of the final step in the electron transport chain, catalyzed by cytochrome *c* and complex IV (COX), is essential for cells to efficiently generate ATP while minimizing ROS production under normal physiological conditions (Figure 15A). In detail, complex IV catalyzes the transfer of electrons to molecular oxygen (O_2_). Its reduction to two water molecules requires four electrons transferred from cytochrome *c*, together with four protons, which are taken from the mitochondrial matrix.

**Figure 14:**
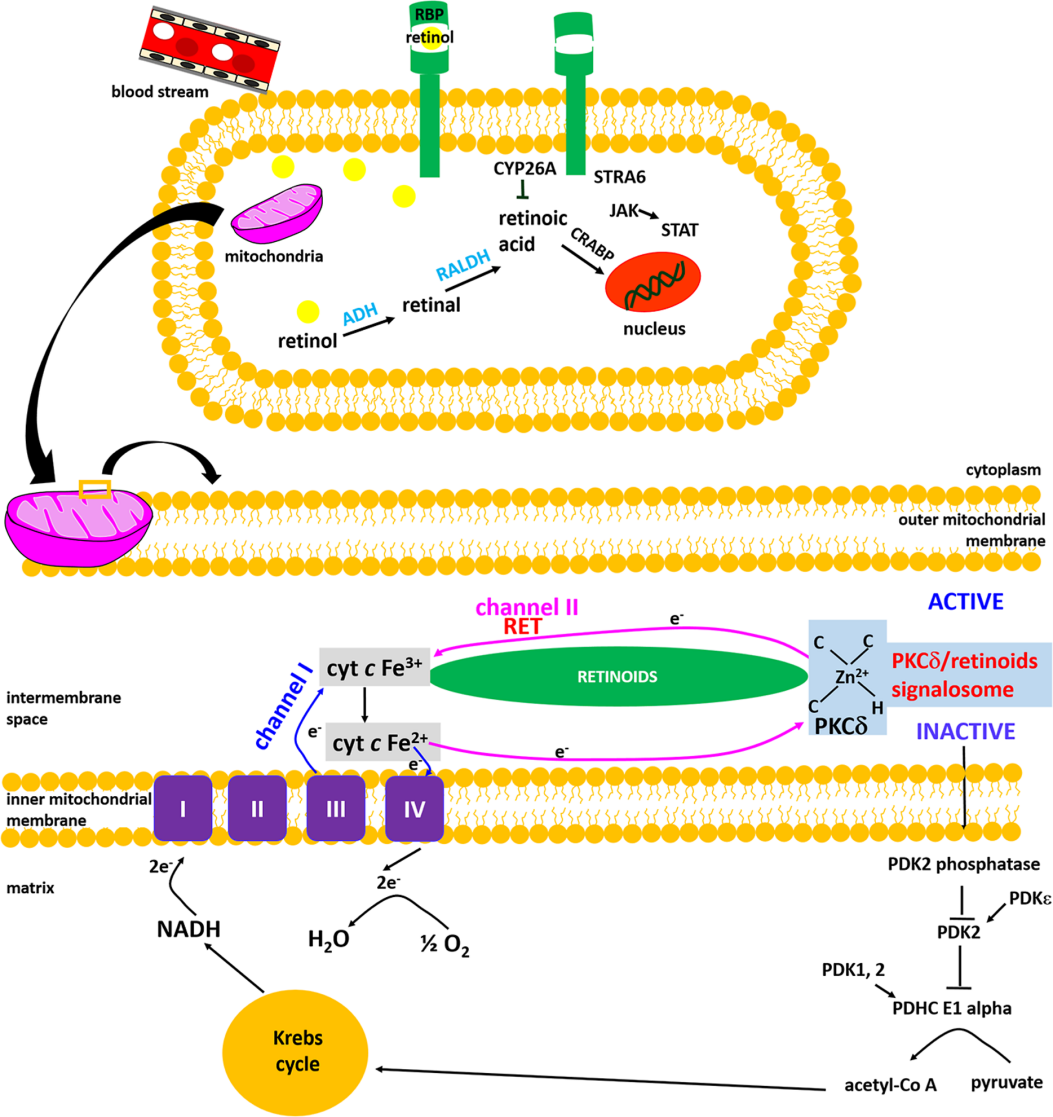
Mechanism of redox status regulation of cytochrome *c* by the PKCδ/retinol signalosome, located in the mitochondrial intermembrane space.

**Figure 15:**
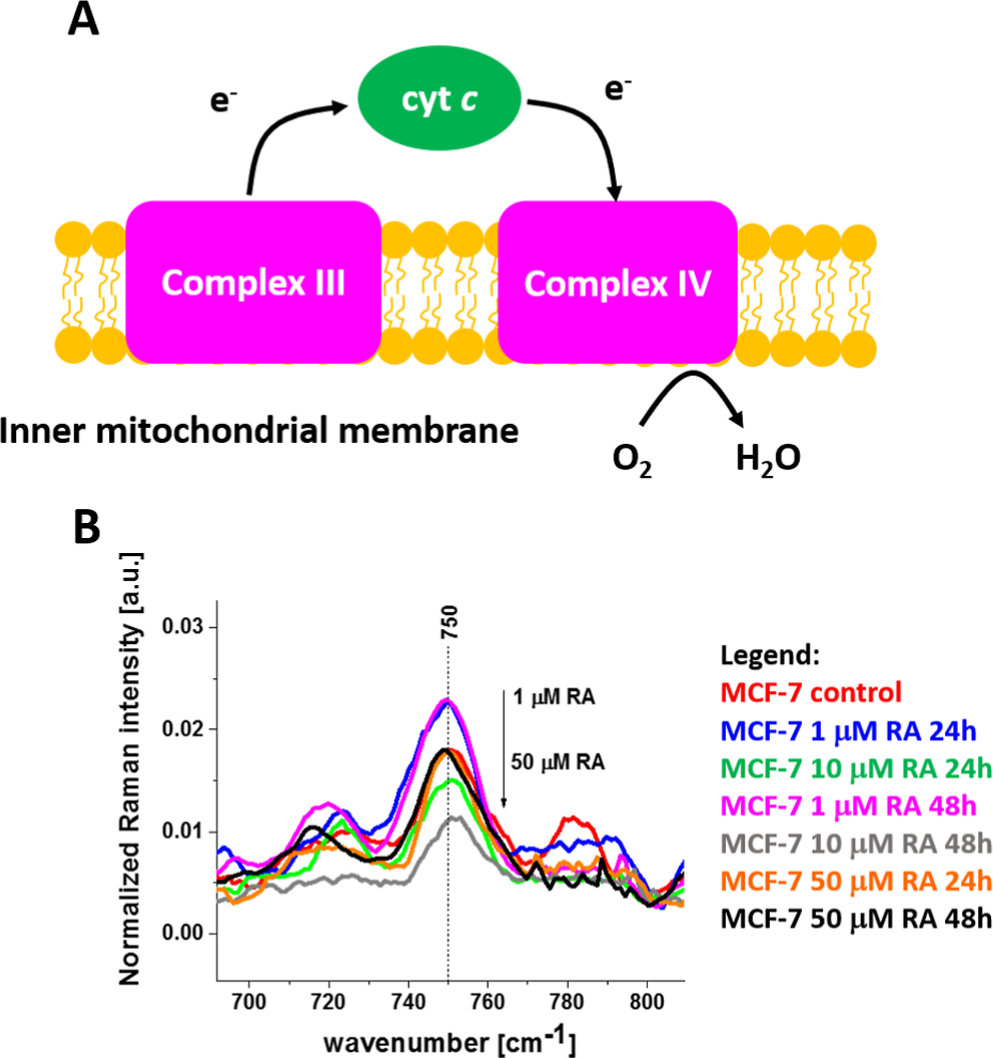
(A) The electron transfer from complex III to complex IV by cytochrome *c*, (B) normalized average Raman spectra in the range 700–800 cm^–1^ for the mitochondria of human breast cancer cells (MCF-7) without (control) and incubated with retinoic acid at a concentration of 1, 10, and 50 μM for 24 and 48 h.

Our results provide evidence that the electron transfer from cytochrome *c* to complex IV (COX) at the terminal step of the ETC is the rate-limiting step of this vital process in cellular bioenergetics. Indeed, Figure 15B shows the Raman signal for the mixed vibration corresponding to cytochrome *b* (in complex III) and cytochrome *c* in the ETC of mitochondria. In contrast to the results presented in Figure 4E for the Raman intensity of pure cytochrome *c* vibration at 1582 cm^–1^, the intensity at 750 cm^–1^ representing complex III decreases with the retinoic acid concentration. It indicated that the electron flux between complex III and cytochrome *c* is physiologically correct with an increase of Fe^2+^ form of cytochrome *c* accompanied by a decrease (more oxidized form Fe^3+^) in complex III. Therefore, the bottleneck effect for electron flux must occur between cytochrome *c* and complex IV. The mechanism of the bottleneck between cytochrome *c* and complex IV is still unknown. Our results from this paper on decreasing tyrosine activity upon retinoic acid (Figures 5 and 8) suggest that reversible phosphorylation of cytochrome *c* mediated by cell signaling pathways are primary regulatory mechanisms of the last step of the ETC in human breast cancer cells of MCF-7. The effects of cytochrome *c* phosphorylation on ETC and apoptotic function have far-reaching implications. Blocking of the electron flux between cytochrome *c* and complex IV determines gradient ΔΨ_m_, ATP production, and ROS generation, linking oxidative phosphorylation to human cancer through a lack of energy, ROS production, cytochrome *c* release, and the activation of apoptosis. Under normal physiological conditions, cytochrome *c* is phosphorylated in an optimal manner to control the ETC flux, ΔΨ_m_, ATP production, and ROS generation. Phosphorylated cytochrome *c* is less likely to be released from the mitochondria to activate apoptosis in the cytosol.

The recent identification of new phosphorylation sites on cytochrome *c* has deepened our understanding of its essential role in controlling ETC flux and thus mitochondrial respiration, ΔΨ_m_, ATP, and ROS.^54–59^

This paper provided experimental evidence that retinoic acid affects the redox status of cytochrome *c* that can decisively affect ETC flux in intact cells in addition to regulating apoptosis. We found that phosphorylation of cytochrome *c* plays a crucial role in regulating the redox status of cytochrome *c* in mitochondria. To summarize, we showed that retinoic acid blocks the flux between cytochrome *c* and complex IV in conventional channel I in ETC.

To explain how mitochondria regulate cytochrome *c* activities upon retinoic acid in cells, we recently proposed^10^ the existence of the second channel (channel II in Figure 15) based on the quantum chemistry models of PKCδ-retinoic acid complex catalysis resonance energy transfer (RET) reactions.^60^ At normal physiological conditions and at low concentration of retinoic acid, the overload of reduced Fe^2+^ cytochrome *c* is regulated by interplay between the channel I and channel II. Briefly, we proposed how retinoic acid catalyzes resonance energy transfer (RET) reactions.^10^ We suggest that mitochondrial energy homeostasis critically depends on the RET-driven regulation of oxidative phosphorylation, with PKCδ identified as the key regulatory protein in this mechanism. PKCδ triggers a signal for the pyruvate dehydrogenase complex. The PKCδ−retinoic acid complex reversibly responds to the redox potential of cytochrome *c* that regulates the electron transfer chain flux (Figure 14). The details of the RET mechanism are explained in our recent paper.^10^

We suggest that the process of reversible redox regulation of the PKCδ/retinoic acid (and to a lesser extent PKCδ/retinol) signalosome by the coupling between channels I and II occurs only at normal physiological conditions. During cancer development, resonance energy transfer (RET) leads to the irreversible activation of PKCδ. As a result, although retinoids can initiate the exergonic activation pathway, they are unable to engage the endergonic silencing pathway, leaving PKCδ locked in the active state and leading to the excessive production of reactive oxygen species (ROS).

This theoretical hypothesis presented in ref 61 received experimental preliminary support from our results presented in the recent paper.^10^ The results presented in this paper enhanced even more significantly the theoretical mechanism of RET. The results of this paper show the enhanced production of reduced Fe^2+^ cytochrome *c* in MCF7 cancer cells. The enhanced electron flux to cytochrome *c* from unbalanced PKCδ/retinoid molecule signalosome increases spectacularly the Raman signal of the reduced cytochrome in cancer cells because the reduced cytochrome (Fe^2+^) has much higher intensity of the Raman bands than the oxidized cytochrome *c* (Fe^3+^).^62^ Consequently, the increase in Raman signal at 1582 cm^–1^ upon incubation with retinoic acid demonstrates a conversion from oxidized cytochrome *c* to reduced cytochrome *c*.

The transition from reversible to irreversible RET mechanisms in cancer remains poorly understood; however, we propose that it may depend on the enzymatic balance, as illustrated in Figure 2B.

When the Krebs cycle is deactivated by retinoic acid, the acetyl CoA cannot be metabolized by the Krebs cycle, and the reaction of reduction of NAD^+^ to NADH (and FAD to FADH_2_) is blocked and cannot produce high-energy electrons from NADH (and FADH_2_). The limited flux of electrons along the electron-transport chain in the inner membrane of the mitochondrion cannot generate a proton gradient across the inner membrane, which is used to trigger the production of ATP by ATP synthase. When the Krebs cycle is deactivated by retinol, the cell switches to a channel of glycolysis in the cytosol, which is less favorable in terms of energy storage, but it is much faster. The RET mechanism also explains the increased production of lipid droplets filled with TAGs that we observe in breast cancer cells (Figure 10). Inside the mitochondria, there are enzymes that metabolize pyruvate and fatty acids to produce acetyl CoA that can be used for enhanced synthesis of fatty acids stored in lipid droplets when the flux of pyruvate into the Krebs cycle is reduced. Therefore, this RET mechanism explains the enhanced de novo synthesis of lipids observed in the cancer cells incubated with retinoic acid. At normal physiological conditions, the lipid droplets are predominantly filled with retinyl esters resulting in lower concentration of retinol in cytosol needed for normal functioning. In cancer cells incubated with retinoic acid, CoA is used for enhanced fatty acid de novo synthesis and their storage in TAG lipid droplets.

## 4 Conclusions

Given the current understanding of retinoid effects on cancer, our study shows that retinoids modulate both the redox state of cytochrome *c* and tyrosine kinase activity in triple-positive human breast cancer cells (MCF-7). We showed that Raman imaging can be used as an effective assay for detecting the redox status of cytochrome *c* and tyrosine kinase activity in specific cell organelles upon incubation with retinoic acid. Compared with existing analytical techniques, Raman imaging uniquely captures the complete distribution of cytochrome *c* and tyrosine activity inside and outside specific organelles.

We studied the impact of retinoids on the redox status of the central iron ion in the heme of cytochrome *c* in triple-positive human breast cancer cells (MCF-7). We recorded the Raman spectra and images in human breast cancer in vitro MCF-7 cells receiving redox stimuli by retinoic acid. We incubated human breast cancer cells (MCF-7) with retinoic acid at concentrations of 1, 10, and 50 μM for 24 and 48 h of incubation. We determined the redox status of cytochrome *c* in mitochondria, cytoplasm, membrane, lipid droplets, and endoplasmic reticulum. We discuss the role of retinoic acid in modifications of oxidative phosphorylation and signaling in cancer cells. Our findings demonstrated that retinoic acid plays a crucial role in maintaining mitochondrial energy homeostasis by regulating the redox state of cytochrome *c* within the electron transport chain that results in effectiveness of oxidative phosphorylation and apoptosis. The results based on the Raman vibrational landscape presented in this paper explain the interplay between the signaling and metabolic pathways in cancer development, demonstrating how redox status of cytochrome *c* and tyrosine kinase activity are modified by retinoic acid. The balance between Fe^3+^/Fe^2+^ heme forms of cytochrome *c* is spectacularly shifted toward the reduced form Fe^2+^ upon retinoic acid presence in a cell. This shift has very serious consequences for regulating overall electron transport chain flux, proton gradient ΔΨ_m_, ATP production, and ROS generation and cytokine induction. We found that retinoic acid spectacularly decreases mitochondrial respiration and tyrosine activity.^10^ These events were monitored by the Raman signals at 1582 cm^–1^, 1616 cm^–1^, 3058 cm^–1^, and 3072 cm^–1^ in the mitochondria, cytoplasm, lipid droplets, endoplasmic reticulum, and nucleus. The paper provides experimental support for theoretical hypothesis how retinoic acid regulates the flux of electrons in the electron transport chain by the coupling with the resonance energy transfer reactions (RET) that control the activation/inactivation cycle of protein kinase PKCδ.

Reversible phosphorylation of cytochrome *c*, regulated by cell signaling pathways in conjunction with RET, is proposed as a key mechanism controlling mitochondrial respiration, electron transport chain (ETC) activity, proton gradient (ΔΨm), ATP synthesis, and ROS generation—linking oxidative phosphorylation to cancer via energy deficits, ROS production, cytochrome *c* release, and apoptosis activation.

## 5 Materials and Methods

### 5.1 Reference Chemicals

Tyrosine (no. T3754, purity HPLC ≥98%), phosphotyrosine (no. P9405, purity HPLC ≥98%), cytochrome *c* (no. C2506, purity SDS-PAGE ≥95%) and retinoic acid (no. R2625, purity HPLC ≥98%), retinol (no. R7632, purity HPLC ≥95%), retinyl palmitate (no. R3375, purity HPLC ≥90%), all-trans-retinal (no. R2500, purity HPLC ≥98%), and retinol binding protein (no. R9388, purity SDS-PAGE ≥85%) were purchased from Merck Life Science.

### 5.2 Cell Culture and Preparation for Raman Spectroscopy and Imaging

MCF7 (HTB-22, ATCC) cells were cultured according to ATCC protocol in the EMEM (Eagle’s Minimum Essential Medium, ATCC 30-2003), supplemented with 0.01 mg/mL bovine insulin (Sigma-Aldrich, St. Louis, MO, USA) and 10% fetal bovine serum (FBS, ATCC 30-2020). Cells were grown at 37 °C under a humidified atmosphere of 5% CO_2_. For Raman imaging, cells were supplemented with 1, 10, and 50 μM of retinoic acid by 24 and 48 h. After Raman imaging cells were stained with Hoechst 33342 (25 μL at 1 μg/mL in PBS) and Oil Red O (10 μL of 0.5 mM in 60% isopropanol/dH_2_O per mL of PBS), followed by a 15 min incubation. Imaging was carried out using 355 nm excitation for Hoechst and 532 nm for Oil Red O, each with an integration time of 0.01 s and spatial resolution of 1 μm. Following a PBS wash, the cells were imaged for fluorescence using an Alpha 300RSA WITec microscope with the addition of fresh PBS.

### 5.3 Raman Spectroscopy and Imaging

Raman measurements of the human breast adenocarcinoma were conducted on a WITec confocal alpha 300 Raman microscope with the use of a 532 nm excitation wavelength coupled to the microscope via an optical fiber (50 μm diameter). A Zeiss objective with a magnification of 40× and a numerical aperture (NA = 1.0) intended for cell measurements performed by immersion in PBS have been used. Before acquisition of the Raman spectra, a standard single-point calibration was conducted using the characteristic Raman peak of a silicon plate at 520.7 cm^–1^. The spectra were recorded with a 532 nm excitation laser power of 10 mW and with an integration time of 0.3 s by an Andor Newton DU970-UVB-353 CCD camera in enhanced mode (EMCCD). Raman data analysis was carried out using WITec (WITec Project Plus 4) and OriginPro 2024 programs. Raman imaging data were analyzed by the cluster analysis method described in.^8,9,15,16^ Raman maps presented in the manuscript were constructed based on principles of cluster analysis described in detail in.^8,9,15,16^ The data set was divided into six clusters—the minimum number required to distinguish unique average Raman spectra associated with organelles: nucleus, lipid droplets/ER, cytoplasm, mitochondria, and membrane. Cluster colors are consistent with those used in the Raman spectra, indicating specific cellular components: red for nucleus, orange for lipid droplets, blue for endoplasmic reticulum, green for cytoplasm, magenta for mitochondria, and light gray for the cell border.

### 5.4 Statistical Analysis

All results regarding the analysis of the intensity of the Raman spectra of breast cancer as a function of retinoic acid concentration are presented as the mean ± SD, where *p* < 0.05 (SD-standard deviation, p-probability value). The average Raman spectra were calculated based on the number of spectra given below. Number of analyzed cells *n*(MCF-7) = 4, *n*(MCF-7 with 1 μM retinoic acid) = 6, *n*(MCF-7 with 10 μM retinoic acid) = 7, *n*(MCF-7 with 50 μM retinoic acid) = 8; number of control and incubated with retinoic acid Raman spectra of MCF-7 used for averaging 7424, 14811, 15943, and 12588, respectively. The average normalized Raman spectra were normalized by vector norm.

### 5.5 ANOVA

Spectroscopic data were statistically analyzed via a one-way ANOVA test using OriginPro 2016. Statistical significance was determined through the Tukey test; results marked with an asterisk indicate p-values ≤0.05.

## Data Availability

The data underlying this study are openly available in RDB at https://rdb.p.lodz.pl/dataset.xhtml?persistentId=doi:10.34658/RDB.SZDIZG.
